# Parachuting Behavior and Predation by Ants in the Nettle Caterpillar, *Scopelodes* *contracta*

**DOI:** 10.1673/031.010.3901

**Published:** 2010-04-27

**Authors:** Kazuo Yamazaki

**Affiliations:** Osaka City Institute of Public Health and Environmental Sciences, 8-34 Tojo-cho, Tennoji, Osaka 543-0026, Japan

**Keywords:** descending behavior, larval movement, pupation site, slug caterpillar

## Abstract

This paper documents the bizarre descending behavior from the tree crown to the ground of the larvae of the moth, *Scopelodes contracta* Walker (Lepidoptera: Limacodidae) and the interaction of the larva with predatory ants. *S. contracta* larvae infest leaves of many tree species in urban areas and orchards in Japan. Mature larvae and leaves without basal leaf parts were found under trees of four species infested with *S. contracta* larvae in Osaka, Japan. Individual larvae riding on leaves were observed falling from tree crowns to the ground. Many *S. contracta* cocoons were found in the soil below the trees two weeks after the observed parachuting. These observations indicate that *S. contracta* larvae parachuted to the ground where they spin their cocoons in the soil. When a larva that had just parachuted down was returned to an arboreal twig, the larva repeated the parachuting behavior. This parachuting behavior appears to be adaptive, because larvae can descend to the ground safely and with low energy cost. Worker ants of *Tetramorium tsushimae* Emery (Hymenoptera: Formicidae) and *Pristomyrmex punctatus* Mayr (Hymenoptera: Formicidae) occasionally attacked larvae on the ground before they had a chance to burrow in the soil.

## Introduction

Mature Lepidoptera larvae seek diverse, species-specific sites suitable for pupation, such as in the soil, between leaves, under bark, and on tree trunks. This behavior may be favorable for them, by avoiding enemies or the adverse microclimate (Odebiyi et al. 2003; Veldtman et al. 2007). As many lepidopteran species pupate near the ground, including in soil or leaf litter, arboreal caterpillars likely descend from their food plants to the ground for pupation. They usually crawl, descend on silk threads, or occasionally fall to the ground (e.g., Shirota et al. 1976; Sun et al. 2000; Wang et al. 2000). However, these methods of descent may not be safe or involve energy expenditure. Wandering long distances could expose caterpillars to many predators and parasitoids. Producing silk threads requires protein and energy (Berenbaum et al. 1993), and falling from tree canopies directly to the ground may inflict mechanical damage to larvae (cf. Yanoviak et al. 2008). In this paper, an efficient descent behavior of the nettle caterpillar, *Scopelodes contracta* Walker (Lepidoptera: Limacodidae), and its interaction with important natural enemies are described.

*S. contracta* is widely distributed and is found in Japan, China, and India and infests various tree species such as persimmon, bayberry, and cherries (Sakagami et al. 2003). Although the population density of *S. contracta* is usually relatively low, this moth sometimes becomes epidemic in urban areas and orchards. Eggs are laid in batches on the underside of host leaves, and hatched larvae feed on the leaves in groups as early instars and solitarily as late instars. The larvae, which are slug-like and up to ∼25 mm long, emerge twice a year (June to July and August to October) in central Japan (Sakagami et al. 2003; Yamazaki personal observations). Miyatake (1977) briefly noted that mature *S. contracta* larvae riding singly on leaves fell to the ground from a hackberry tree, *Celtis sinensis* Persoon (Rosales: Ulmaceae), to pupate in the soil. However, this bizarre behavior has yet to be examined in more detail, and other aspects of the larval ecology of this species have rarely been studied. In the present study, the descent behavior of mature *S. contracta* larvae and the associated attacks by ants were examined to improve the understanding of the natural history of this moth.

## Materials and Methods

### Study sites

Field studies were conducted at three sites: Tsurumi-ryokuchi Park (34° 43′ N, 135° 35′ E, ∼5 MASL), Shotenyama Park (34° 38′ N, 135° 30′ E, ∼7 MASL), and the bank of the Yamato River (34° 35′ N, 135° 30′ E, ∼5 MASL) in Osaka Prefecture, central Japan. These sites were located in the urban area of Osaka. At the two parks, many trees, including those examined in the study, had been planted, while naturally occurring willows (*Salix* spp.) and *C. sinensis* grew sparsely along the riverbank.

### Descent behavior

The descent behavior of second-generation *S. contracta* larvae was examined along the bank of the Yamato River and in the Tsurumiryokuchi Park in autumn 2008. On the riverbank, the number of leaves cut by larvae and found on the ground, the number of larvae on the ground, and the number of descending larvae in a 30 min period were counted in a 16 m^2^ (4 m × 4 m) quadrat under a *C. sinensis* tree (tree height: ∼8 m, diameter at breast height: 336 mm) that was clearly suffering from severe herbivory by *S. contracta* larvae. This monitoring occurred during the day (15:00 – 17:00) at 1 to 6-day intervals from late September to mid-October. Larvae on tree foliage were carefully observed in order to witness the cutting of the basal part of the leaves for parachuting. In addition, an *S. contracta* larva was returned to an arboreal leaf (∼1.3 m above the ground) soon after it parachuted to the ground, and its behavior was observed at 16:25 on 5 October. To survey *S. contracta* individuals in the soil, ten 0.09 m^2^ (0.3 m × 0.3 m) quadrats were established in arbitrarily chosen locations under the *C. sinensis* tree, and they were dug to a depth of 50 mm using a hoe to inspect moth cocoons. New cocoons could be discriminated from older ones because of the light color and lack of emergence holes.

At Tsurumi-ryokuchi Park, the number of larval-cut leaves found on the ground, the number of larvae on the ground, and the number of larvae descending in a 30 min period were counted under a tree of heaven, *Ailanthus altissima* Swingle (Sapindales: Simaroubaceae), (tree height: ∼15 m) on 2 October. In addition, one yoshino cherry, *Prunus* × *yedoensis* Matsumura (Rosales: Rosaceae) (∼7 m tall) and three keyaki *Zelkova serrata* (Thunb.) Makino (Rosales: Ulmaceae) (∼7 m tall) were inspected for descent behavior by *S. contracta* larvae. Arboreal larvae were observed carefully for 5 h, as described above, to document their leafcutting behavior.

**Table 1.**
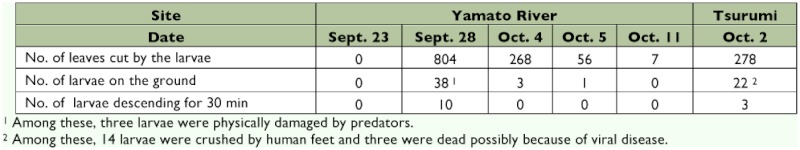
Observations on parachuting behavior of *S. contracta* larvae at two urban sites in cenral Japan.

### Ant predation

Predation by ants on first-generation *S. contracta* larvae was observed at the Tsurumi-ryokuchi and Shotenyama Parks during the day (14:00 – 17:00) on 10 July 1998 and 5 August 2007, respectively. At Tsurumiryokuchi Park, many *S. contracta* larvae were inspected under one poplar, *Populus nigra* var. *italica* L. (Salicales: Salicaceae), (∼10 m tall), that was heavily damaged by the larvae. At Shotenyama Park, six *S. contracta* larvae were observed under a *C. sinensis* (∼5 m tall). Some of the ants attacking the larvae were collected for identification. Attacking ants were also sought during the examination of larval descent behavior.

## Results

### Descent behavior

**Figure 1.**
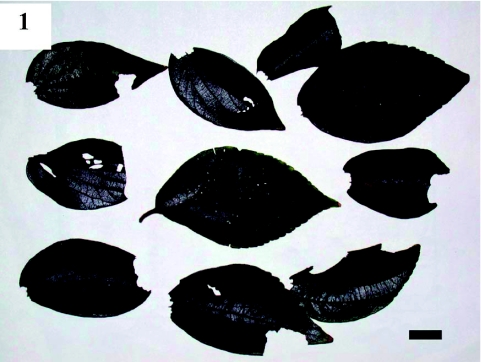
*Celtis sinensis* leaves that were cut by *S. contracta* larvae for parachuting. Basal parts are removed. Scale bar: 10 mm. High quality figures are available online.

Along the riverbank, the *C. sinensis* tree suffered from severe herbivory by a number of *S. contracta* larvae, with a visual estimate of leaf area loss of ∼90%. On 23 September, no descent behavior was observed, while on 28 September, many larvae were seen exhibiting descent behavior (Table 1). On the ground, there were 804 leaves with the basal parts consumed, 35 crawling or motionless larvae, and three dead larvae, possibly killed by predators, were observed. In a 30 min period, 10 larvae were observed singly on leaves falling to the ground (Table 1). All parachuting larvae were on the upper leaf surface. Most leaves lacked most basal parts of the lamina, but some lacked only the petiole (Figure 1). The heads of the parachuting larvae faced the base of the leaf. The larvae began burrowing into the soil to spin their cocoons 1 to 5 min after reaching the ground. Descent behavior was also observed on 4 and 5 October, although it was past its peak. On 11 October, no larvae were found on the ground; only one larva was observed on a twig, and only seven cut leaves were present on the ground (Table 1).

**Figure 2.**
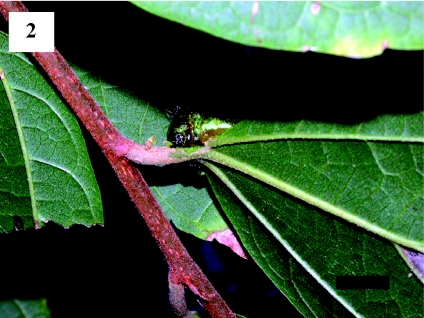
*Scopelodes contracta* larva feeding on the apical part of a petiole. The larva had just parachuted to the ground and was returned to a twig of the study tree. Scale bar: 10 mm. High quality figures are available online.

**Figure 3.**
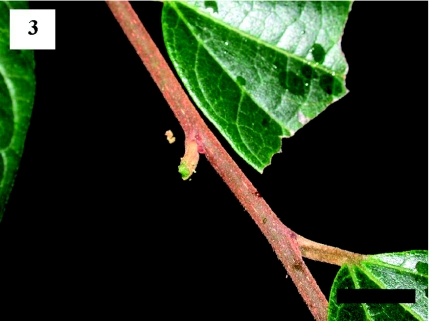
The petiole cut by the *Scopelodes contracta* larva. Scale bar: 10 mm. High quality figures are available online.

**Figure 4.**
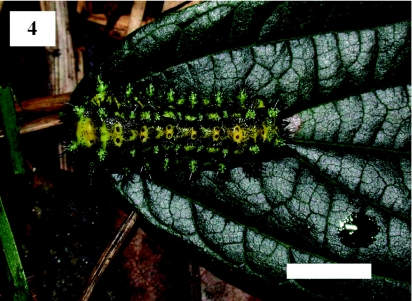
*Scopelodes contracta* larva on a *Celtis sinensis* leaf just after reaching the ground by parachuting. Scale bar: 10 mm. High quality figures are available online.

Although more than 50 *S. contracta* larvae were carefully inspected on arboreal leaves, none were observed cutting the leaf bases. However, when a larva that had just parachuted to the ground was returned to a twig of the study tree, the larva repeated its parachuting behavior (Figures 2, 3, 4). First, the larva spun a few silk threads on the basal part of a leaf and then positioned itself with its head facing the petiole. The larva subsequently fed on the apical part of the petiole (Figure 2). After 11 min, the connection of the petiole to the twig became unstable and the leaf swung from side to side. After 14 min, the larva parachuted to the ground on the falling leaf (Figures 3 and 4).

A number of spherical *S. contracta* cocoons were found at a soil depth of 0 to 30 mm under the study tree (Figure 6). Examination of these cocoons revealed a density of new cocoons, excluding old empty ones, of 98.9 ± 16.9 (mean ± SE) cocoons/m^2^ (*n* = 10 quadrats examined). This value was relatively close to the density of parachuting larvae of 70.9 larvae/m^2^ estimated based on the cumulative number of cut leaves found under the tree (1135 leaves in 16 m^2^).

**Figure 5.**
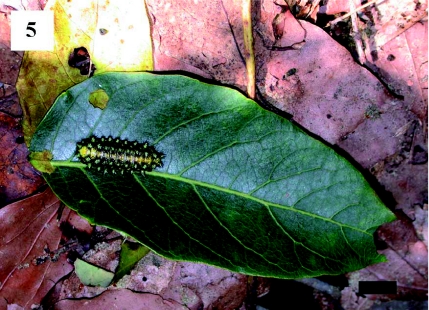
*Scopelodes contracta* larva on an *Ailanthus altissima* leaf just after reaching the ground by parachuting. Scale bar: 10 mm. High quality figures are available online.

At Tsurumi-ryokuchi Park, leaves cut by *S. contracta* larvae and larval parachuting behavior were observed under an *A. altissima* tree (Table 1, Figure 5). In addition, a parachuting larva was found under *a P.* × *yedoensis* tree, and many cut leaves were found under three *Z. serrata* trees. Most leaves cut by larvae were missing basal parts, as in *C sinensis.* No larvae were actually observed cutting leaves on these trees.

### Ant predation

At Tsurumi-ryokuchi Park in July 1998, ∼50 still or crawling *S. contracta* larvae that had just parachuted down to the ground were observed under a *P. nigra* tree. All larvae were attacked, and some were carried away by numerous worker ants, *Tetramorium tsushimae* Emery (Hymenoptera: Formicidae).

**Figure 6.**
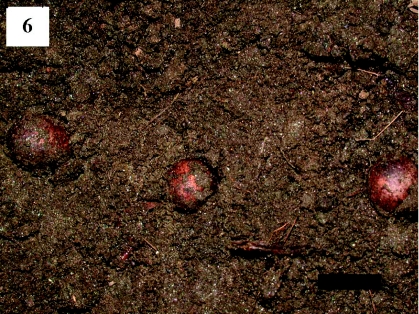
Reddish-brown spherical *Scopelodes contracta* cocoons in the soil. Scale bar: 10 mm. High quality figures are available online.

At Shotenyama Park, six *S. contracta* larvae were attacked by worker ants, *Pristomyrmex punctatus* Mayr (Hymenoptera: Formicidae), under a *C. sinensis* tree. These trees harbored many *S. contracta* larvae. Along the riverbank, and in autumn 2008 at Tsurumi-ryokuchi Park, no ants were observed attacking *S. contracta* larvae.

## Discussion

The presence of fallen leaves without basal parts and crawling larvae, together with the observations of individual larvae on leaves that were falling to the ground (Table 1) of four tree species and the high density of cocoons in the soil (Figure 6), indicate that mature *S. contracta* larvae parachute from tree crowns to the ground to spin their cocoons in the soil. Although the descent behavior has not been sufficiently observed for larvae, the larvae that were preyed on by ants under trees at Tsurumi-ryokuchi and Shotenyama Parks were those that had just descended. Therefore, this study, together with that of Miyatake (1977), suggests that this behavior is an inherent trait of *S. contracta* larvae. This descent behavior can be classified as ‘parachuting,’ because it involves an undirected aerial descent (see Dudley et al. 2007).

However, the consumption of the basal parts of leaves by *S. contracta* larvae was not observed naturally on tree crowns. The low population density of *S. contracta* larvae, their arboreal habits in high tree crowns, and the difficulty in observing limacodid leaf-feeding (due to concealment of the head and mouthparts) lessened the chances of detecting leaf consumption. In addition, leaf cutting may have been completed relatively quickly. In fact, when a larva that had just parachuted to the ground was returned to a twig, the larva cut the apical part of the petiole of another leaf and parachuted back to the ground in 14 min. Under natural conditions, the fall of leaves and larvae may be interrupted midway to the ground by twigs or other leaves; thus, this repeated parachuting behavior may be favorable to the larvae.

In many cases, larvae consumed basal parts of the leaf lamina, and other larvae cut only petioles. Consumption of basal leaf laminas may be more laborious than that of petioles, although the former may contribute more to food intake than the latter. Because *C. sinensis* leaves, a preferred food for the larvae, have a palmate venation with three or four main veins at the basal part of the leaf lamina (Kitamura and Murata 1979, Figures 1, 2, 4), larvae must cut the main veins in order to parachute. However, when a larva cuts a basal leaf lamina in a semicircular manner, the larva can locate itself at the center of the leaf, near the leaf's center of gravity, thereby enabling a more stable descent.

The parachuting behavior of *S. contracta* larvae may be favorable for their cocoon spinning in the soil because both crawling distance and the chance of encountering arboreal enemies may be diminished, and the larvae can descend safely to the ground. This behavior of *S. contracta* may be unique among the speciose Lepidoptera. *Stigmella* sp. (Lepidoptera: Nepticulidae) larvae are leaf miners and induce leaf abscission in their host tree, *Quercus gilva*. They can complete development in the fallen leaves on the ground and pupate in the soil; although, together with the leaves, most are preyed on by deer (Yamazaki and Sugiura 2008). This example is analogous to the present study because both species descend safely from crowns to the ground using leaves. Furthermore, herbivore-caused “greenfall” (non-consumptive leaf loss by herbivores such as petiole clipping, leaf fragment discarding, and leaf abscission) is a widespread phenomenon in forests and may be an important resource for decomposers (Risley and Crossley 1988). Since leaves cut by *S. contracta* contribute to the greenfall, this behavior may affect various biological communities in forests during *S. contracta* outbreaks.

The evolution of this parachuting behavior remains unknown. However, the descent behavior appears to be associated with cocoon spinning in the soil. Limacodids have diverse cocoon-spinning sites, such as among leaves and on twigs or trunks (Young 1986; Sugi 1987; Yamazaki et al. 2007). Limacodid species that make cocoons on twigs or trunks are vulnerable to parasitoid or bird attacks (Yamada 1992; Yamazaki et al. 2007). Although cocoon spinning in soil is not common among limacodids, this habit seems favorable to escape from pupal or prepupal parasitoid and bird attacks. The natural enemies of *S. contracta* have not been intensively examined, except for nuclear polyhedrosis during the larval period (Aratake and Watanabe 1973). In the present study, however, the parachuting *S. contracta* were occasionally, but severely, attacked by numerous ants just after landing. If ant colonies are located under host trees, upon descent, most larvae may be preyed on by ants, as in July 1998 at Tsurumi-ryokuchi and in August 2007 at Shotenyama Park. As the *C. sinensis* tree along the riverbank was located within 5 m of flowing river water, most larvae there may successfully spin cocoons in the soil because of the ant-poor environment. The relationship between the unique parachuting behavior and ant predation of *S. contracta* larvae may account for the fluctuating population dynamics of this species, although further studies on its population ecology, including other developmental stages and mortality factors, are required.
